# UAV sensor failures dataset: Biomisa arducopter sensory critique (BASiC)

**DOI:** 10.1016/j.dib.2024.110069

**Published:** 2024-01-15

**Authors:** Muhammad Waqas Ahmad, Muhammad Usman Akram

**Affiliations:** Department of Computer and Software Engineering, College of Electrical and Mechanical Engineering, National University of Sciences and Technology (NUST), Islamabad, 44000, Pakistan

**Keywords:** Sensors failures dataset, Autonomous flights, ArduPilot, Unmanned aerial vehicle, Autopilot, Mission planner, Software in the loop, UAV

## Abstract

Unmanned aerial vehicles (UAV) rely on a variety of sensors to perceive and navigate their airborne environment with precision. The autopilot software interprets this sensory data, acting as the control mechanism for autonomous flights. As UAVs are exposed to physical environment, they are vulnerable to potential impairments in their sensory mechanism. Their real-time interactions with the actual atmosphere make them susceptible to cyber exploitations as well, where sensory data alterations through counterfeit wireless signals pose a significant threat. In this context, sensor failures can result into unsafe flight conditions, as the fault handling logic may fail to anticipate the context of the issue, allowing autopilot to execute operations without necessary adjustments. Untimely control of sensor failures can result in mid-air collisions or crashes. To address these challenges, we created Biomisa Arducopter Sensory Critique (BASiC) dataset, a state-of-the-art resource for UAV sensor failure analysis. The BASiC dataset comprises 70 autonomous flight data, spanning over 7 hours. It encompasses 3+ hours of (each) pre-failure and post-failure data, along with 1+ hour of no-failure data. We selected the ArduPilot platform as our demonstration aerial vehicle to conduct the experiments. By engineering Software in the Loop (SITL) parameters, we effectively executed sensor failure test simulations. Our dataset incorporates six representative sensors failures which are critical to UAV operations: global positioning system (GPS) for precise aerial positioning, remote control for communication with the ground control station (GCS), accelerometer for measuring linear acceleration, gyroscope for rotational acceleration measurement, compass providing heading information, and barometer for maintaining flight height based on atmospheric pressure data. The availability of the BASiC dataset will benefit the research community, empowering researchers to explore and experiment with state-of-the-art deep learning models by tailoring them for time series signal analysis. It may also contribute in enhancing the safety and reliability of mission-critical autonomous UAV flights.

Specifications Table

**Table:** 

Subject	Aerospace Engineering
Specific subject area	Timely prediction, detection, and classification of in-flight data-driven failures of autonomous aerial vehicles using various sensory data analytics
Data format	Raw and Filtered
Type of data	Table
Data collection	The simulations involve manipulating the flight dynamics model of the Arducopter to replicate the physics of the UAV. Control inputs from the SITL running the Arducopter firmware are used to adjust and regulate the movements of servos/ motors, and the simulation outputs the aerial vehicle's position, status, velocities, and other flight dynamics. These outputs are then fed back to the control loop of the firmware, replicating the interactions observed during real-world test flights between sensors, components of the plant, and actuators. The pilot controls are executed through MAVLink commands from the ground control station of Mission Planner, facilitated by MAVProxy.
Data source location	**Institution:** BioMedical Image and Signal Analysis (BIOMISA) research group, Department of Computer and Software Engineering, College of Electrical and Mechanical Engineering, National University of Sciences and Technology (NUST). **City:** Rawalpindi and Islamabad **Country:** Pakistan
Data accessibility	**Repository name:** Biomisa Arducopter Sensory Critique (BASiC) Dataset Data identification number: 10.5281/zenodo.8195068 Direct URL to data: https://doi.org/10.5281/zenodo.8195068 Instructions for accessing these data: https://zenodo.org/
Related research article	**Nil**

## 1 Value of the Data

• **Pioneering UAV Sensor Failures Dataset:** In the absence of existing datasets for sensor failures in autonomous aerial vehicles, the Biomisa Arducopter Sensory Critique (BASiC) dataset stands as a novel effort. This dataset, comprising 70 autonomous flight data with over 7 hours of flight time, specifically addresses the lack of comprehensive datasets focused on UAV sensor failures.
• **Diversity in Sensor Failure Scenarios:** The BASiC dataset encompasses six distinct sensor failure types, covering global positioning system (GPS), remote control, accelerometer, gyroscope, compass, and barometer. This diversity provides researchers with a broad spectrum of failure scenarios to analyze, contributing to a more comprehensive understanding of sensor failures in autonomous aerial vehicles.
• **Versatility for AI and Deep Learning Applications:** Researchers, particularly those in the field of artificial intelligence (AI) and deep learning, can benefit from the BASiC dataset. It serves as a resource for advancing sensor failure analysis, allowing for the validation of AI and deep learning algorithms. Researchers can benchmark their models against ground truth information, conduct comparative studies, and develop innovative methodologies, thereby contributing to the improvement of UAV safety and reliability.
• **Contextualizing in Research Landscape:** While the dataset is simulation-based, it significantly expands the scale of available data for sensor faults. In comparison to existing datasets, such as those focused on UAV actuator faults or limited real-flight sensor faults, the BASiC dataset provides a larger-scale simulation dataset. By offering a broader scope, it serves as a foundational resource for early-stage research in UAV sensor fault detection.
• **Potential for Real-World Application:** While acknowledging the limitations of simulation, the BASiC dataset opens avenues for real-world application development. Researchers can use this dataset as a starting point for algorithmic validation and refinement, eventually bridging the gap between simulated scenarios and practical, real-world UAV operations.

## 2 Data Description

The Biomisa Arducopter Sensory Critique (BASiC) [1] dataset contains raw data logs and telemetry logs, capturing flight data in the native formats of ArduPilot and Mission Planner. The raw data logs are recorded within the onboard memory of the Arducopter firmware, while the telemetry logs are subsequently MAVLinked to the ground control station (GCS). These logs serve as evidence of originality, ensuring the replication of flights, including failure scenarios, during subsequent developmental stages. The dataset further includes dataflash binary logs, text logs, and MATLAB's mat files, with all logged messages. FMT (format) sequences in the top rows define message headers, subsequent rows declare the pre-set values of all flight parameters, and the remaining rows contain logged data. Processed data features multiple CSV files elaborating on specific message types and time-serialized and interpolated CSV files consolidating entire flight data.

Each message in the dataset comprises context-specific attributes, providing a comprehensive view of UAV flight data. For example, the GPS message includes attributes such as time, instance number, status, and number of visible satellites, precision, latitude, longitude, altitude, and speed. Specific indicators adapt values in response to failures during flight, providing insight into the UAV's condition. The dataset also includes illustrative data of Table 1, showcasing symptomatic messages, corresponding symbolic attributes, pre-failure default values, and post-failure indicative flags.

**Table 1:** Sensor failure status messages and associated parametric values.

No.	Sensor	Message	Parameter	Pre-Failure	Post-Failure
1	GPS	GPS	Status	6	1
2	Remote Control	MODE	Mode	Auto	RTL
3	Accelerometer	IMU	AH	1	0
4	Gyroscope	IMU	GH	1	0
5	Compass	MAG	Health	1	0
6	Barometer	BARO	Health	1	0

In the processed data, each message type is represented by a distinct CSV file with enhanced attribute names. Attribute standardization introduces a unified *“Status”* attribute, transitioning from 0 to 1 when any failure occurs during flight. This data is time-indexed, missing values are interpolated, and columns are structured systematically, making it ready for experimenting with advanced data-driven deep learning techniques in UAV sensor failure analysis.

### 2.1 Directory structure

**Fig. 1:**
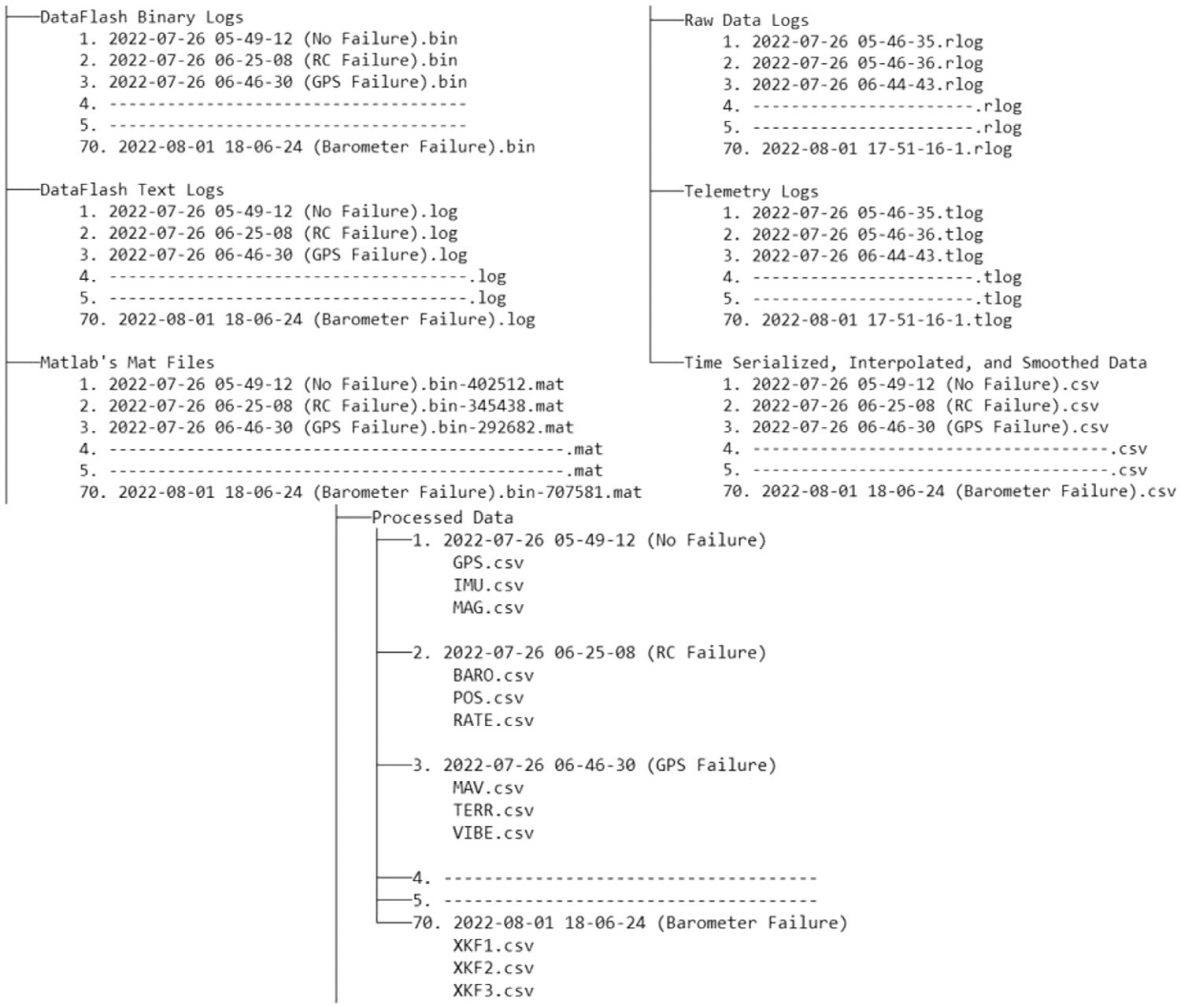
The BASiC dataset exhibits a well-structured directory and file organization, including raw data logs, telemetry logs, dataflash binary logs, text logs, Matlab files, and processed data.

The BASiC dataset's directory structure is illustrated in Fig. 1, showcasing a methodical and well-organized approach. Each test flight is uniquely identified with a coded name in the format of YYYY-MM-DD hh-mm-ss (Failure Type), highlighting the precise date and time of the flight, along with the specific type of failure explored. In the absence of failure, it is indicated as (No Failure). This naming convention ensures clear identification and efficient navigation through the dataset. The dataset encompasses various data formats, including extensions such as bin, log, mat, rlog, tlog, and CSV. It is carefully designed to incorporate six distinct types of data formats, each serving a specific purpose and contributing to the dataset's versatility and richness.

### 2.2 Data formats

The dataset is comprised of following six types of data formats:

### 2.3 Dataflash binary logs

Dataflash binary logs serve as the primary source for post-flight analysis, recorded in the onboard memory of Arducopter firmware without any pre-processing. These logs initiate recording shortly after the aerial vehicle is armed and capture comprehensive flight data until landing. Presented as CSV files in binary format, each line uniquely numbered and the file's top section displays the software version and firmware board type. FMT messages follow, providing essential metadata that outlines column headers, data types, and format-related information for various message types. Subsequently, the PARM sequences define all parameters alongside their default values. The dataflash binary logs then record extensive flight data messages for various modules, including *GPS, MAV* for remote control, *IMU* for accelerometer and gyroscope, *MAG* for compass, and *BARO* for barometer, among others. To fully unlock the insights within these dataflash binary files, commercial off-the-shelf software such as Mission Planner, MAVExplorer, UAV LogViewer, QGroundControl, or Dronee Plotter is essential.

### 2.4 Dataflash text logs

To enhance accessibility and facilitate seamless numerical analysis, we converted the dataflash binary logs into text logs, ensuring compatibility with common text editors and spreadsheet programs. This conversion maintains complete consistency between the binary and text formats, preserving the data's integrity throughout the process. The text logs, in CSV format, encompass FMT messages, PARM messages, and onboard log messages, all presented in a readily readable text layout. Software applications such as Windows Notepad, WordPad, Microsoft Excel, or Gedit for Linux can be used to process the contents of these dataflash text files.

### 2.5 Processed data

The dataflash text logs, prepared for pre-processing, underwent an advanced code script, designed to automate the processing step. With precision and efficiency, the script methodically read through the contents of the text logs, extracting message headers, column names, and data types from the FMT sequences. Each header corresponded to a distinct series of sequences, encapsulating data pertaining to sensors, actuators, servos, motors, plants, and other critical control components. These sequences were carefully segregated and saved as separate CSV files, ensuring optimal organization and easy access. Upon exploring the flight data messages, the script established precise linkages between sequence contents and their respective columns in CSV files. The outcome was a collection of CSV files, each containing specialized tokens derived from the onboard log messages. These decomposed dataflash log files expressed as a series of CSV files with unique spatial dimensions and original timestamps as the first column of each file.

To further enhance data consolidation across the spatial dimensions, a subsequent pre-processing step initiated intelligent transformations aimed at time-serializing the data into a cohesive, single flat table. We addressed probable data sparsity introduced by time-based serialization through data smoothing. Linear interpolations, filled in any missing values, resulting a smooth spatio-temporal dataset. The realization of this refined spatio-temporal data, indexed by time order for each test case, was accomplished using Python code. The result was a structured dataset, offering significant value for further research and development (R&D) endeavours. An automated script for generating the processed data, encompassing separate CSV files for the entire flight, is available upon request.

### 2.6 MATLAB's mat files

In addition to the processed dataset, we have also included MATLAB's mat binary files for each test flight, providing opportunities for direct processing through MATLAB's powerful matrix manipulation operations. Researchers can readily leverage the rich capabilities of MATLAB's environment to efficiently explore and analyse the data in this format. For detailed insights into this format and its functionalities, valuable information is accessible through MATLAB's official resources, empowering researchers with the necessary tools to unlock the full potential of the dataset.

### 2.7 Raw data logs

The researchers' community is also granted access to the original raw format (rlogs) of flight data for all autonomous flights, provided without any pre-processing. This resource encompasses a wealth of information, with certain files housing multiple flight data instances, while others may disperse a single test flight data across multiple rlog files. It ensures that researchers have access to original flight data, enabling a broad spectrum of exploration and analysis possibilities in pursuit of further advancements in the field of UAV sensor failure diagnostics.

### 2.8 Telemetry logs

The telemetry logs, known as tlogs, provide an extensive array of data similar to that found in dataflash logs, but transmitted over the MAVLink telemetry ports during flight. Functioning as real-time recordings of MAVLink telemetry messages sent from the ArduPilot to the ground control station, Mission Planner, these tlogs present actual footage of flight activity. Researchers can readily leverage these telemetry logs to replay the original flights, paving the way for intelligent insights and further advancements in R&D endeavours.

## 3 Experimental Design, Materials and Methods

The sensor suite of the aerial vehicle provides measurement data of the surrounding environment during flight, serving purposes in both flight attitude control and path planning. The real-time execution of the flight dynamic model introduces susceptibility to various physical impairments [2], potentially leading to failures in sensors [3], plant components, and actuator systems of the UAV [4]. Ensuring the smooth execution of UAV flight missions depends on the timely detection and isolation of sensor failures, a task of critical importance [5]. The field has witnessed the emergence of various strategies aimed at addressing sensor failures in autonomous flights, progressing through successive developments. In a comprehensive study, Cartocci et al. [6] conducted a comparative assessment of different sensor fault estimation techniques. They highlighted that prevailing fault diagnostic approaches may not be sufficient for prompt and reliable sensor fault prevention, citing historical incidents in the aviation domain. However, Hua et al. [7] introduced a fault diagnosis and fault-tolerant control system for UAV sensors based on a back-propagation neural network and genetic algorithm.

Several methodologies for introducing sensor faults into UAVs have been devised to assess their operational integrity. Taylor et al. [8] utilized an in-situ model checking approach, inducing sensor failures to evaluate timely rectification. Wen et al. [9] proposed a closed-loop distributed simulation system to introduce various fault scenarios into UAVs. Xu et al. [10] extended the capabilities of diverse fault injection, utilizing the ArduPilot software-in-the-loop platform to simulate sensor faults. Gong et al. [11] designed a hardware-in-the-loop simulation system to generate different fault samples, contributing to UAV failure detection research. Keipour et al. [12] introduced the AirLab Failure and Anomaly (ALFA) dataset, containing various fault types in fixed-wing UAV control surfaces and engine. In our research, we adopted their dataset generation approach to conFig. our Ardupilot-based software-in-the-loop setup.

We hereby introduce the Biomisa Arducopter Sensory Critique (BASiC) dataset, a comprehensive compilation of serialized, interpolated, and smoothed data derived from 70 autonomous flights. Our research encompassed experiments involving six distinct sensor failure scenarios, GPS, remote control, accelerometer, gyroscope, compass, and barometer. Additionally, the dataset includes multiple normal flights without any failure, each serving as a valuable reference for comparison. The dataset features an impressive total flight time of 7 hours and 21 minutes, partitioned into 3 hours and 2 minutes of pre-failure data, 3 hours and 15 minutes of post-failure data, and approximately 1 hour and 4 minutes of flight data without any failures.

**Fig. 2:**
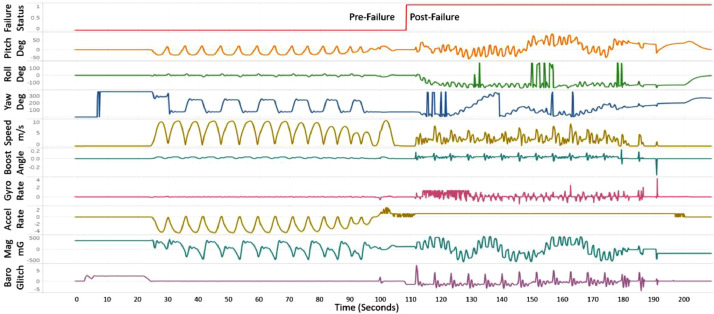
Spatiotemporal signal profile of a random sensor failure test case from the BASiC dataset.

Fig. 2 displays a representative time series signal profile from a random test case in the BASiC dataset. Although the dataset contains over 200 features, for improved visualization, we have presented 10 symbolic features in the figure. The first row includes the failure status label, transitioning from 0 to 1 after failure occurrence. Subsequent rows showcase features such as pitch, roll, yaw, speed, angle, rotational acceleration, linear acceleration, magnetic field strength, and barometer glitches respectively. The varied ranges, along with their corresponding unit of measurement, for each feature are also detailed in Fig. 2, providing an overview of the dataset's signal characteristics.

### 3.1 The platform

The selection of ArduPilot [13] platform for our research endeavour was a natural one, given its widespread recognition in both the academic research community and industry experts. Serving as a versatile, trusted, and open-source autopilot system, ArduPilot accommodates a diverse range of aerial vehicle types, including multi-copters, vertical take-off and landing UAVs, traditional helicopters, and fixed-wing aircraft. Its comprehensive set of tools supports virtually any aerial application, and its firmware can be seamlessly adapted to control various types of UAVs across distinct hardware configurations. In addition to its versatility, the ArduPilot project provides a robust and feature-rich open-source autopilot software for flight dynamics models. To complement our choice of ArduPilot, we selected for the dynamic and full-featured Mission Planner [13] as our ground control station. This open-source application empowers us with precise control over autonomous flights, facilitating seamless interactions with the aerial vehicle during experimental sessions. At the core of our autonomous flights lies in Arducopter [13], multi-copter UAV controller that excels in handling the full spectrum of dynamic flightx requirements.

**Fig. 3:**
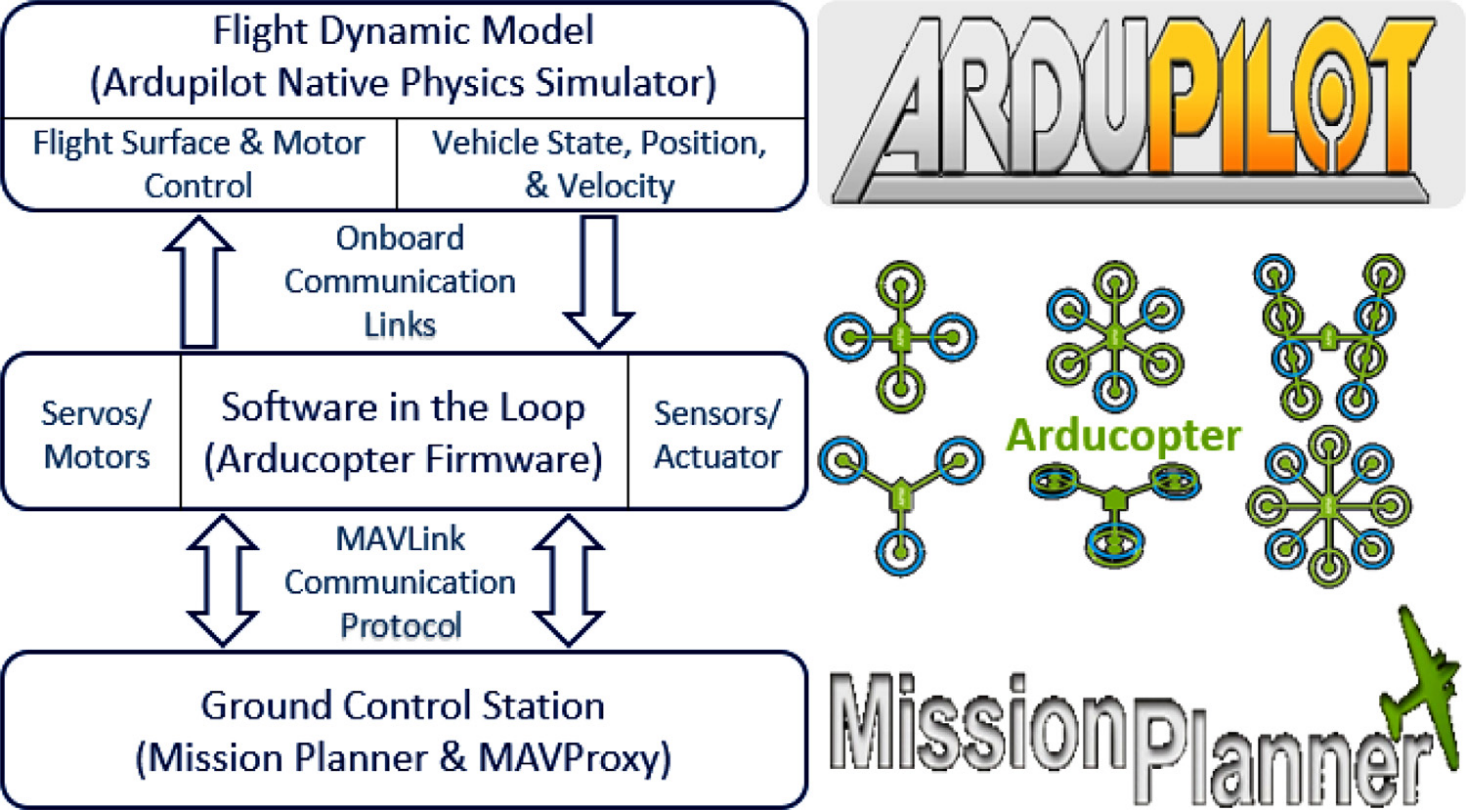
The working model of our experimental setup comprises of Mission Planner and MAVProxy as the GCS, Software in the Loop as the Arducopter firmware controller, and ArduPilot as the native physics flight dynamics model simulator.

Our research involves advanced simulations that manoeuvre the flight dynamics model of Arducopter, replicating the physics of aerial vehicle movement. Synchronized with the SITL (software in the loop) [13] running the Arducopter firmware, our simulations regulate movements of servos and motors, precisely determining the aerial vehicle's position, status, velocities, and other important flight dynamics. These inputs are fed back into the firmware simulation's control loop, accurately mirroring the interactions observed between sensors, components of the plant, and actuators during real-world test flights. Pilot controls during these simulations are executed through MAVLink commands, issued by the Mission Planner and the command-line-based GCS, MAVProxy. The operational dynamics of the experimental platform, encompassing the seamless integration of firmware and software in the loop, are visually depicted in Fig. 3.

**Fig. 4:**
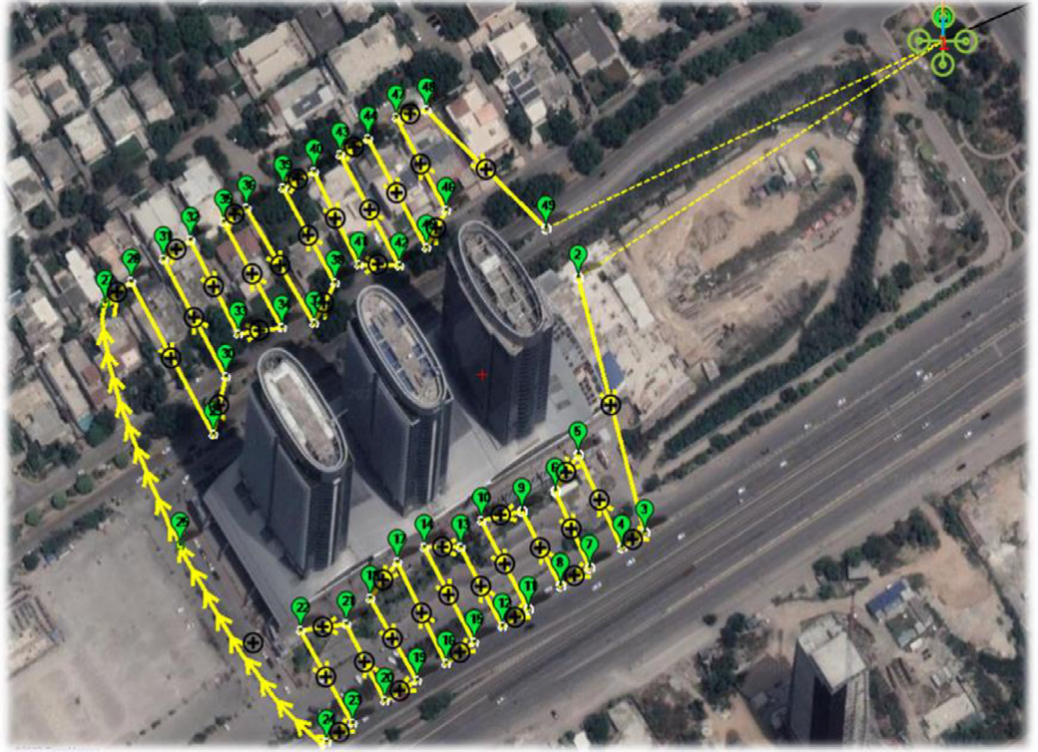
The sample location for the test flight demonstrates the UAV's dynamic capabilities, mission execution, and data recording. It showcases the UAV's position, designated home point, flight trajectory, waypoints, and return-to-launch.

The test flights conducted for this research were undertaken by the BioMedical Image and Signal Analysis (BIOMISA), a research group of academic researchers and industry experts working in close collaboration to provide state-of-the-art solutions for real-world problems [14]. Within Fig. 4, a sample test case is displayed, representing one among the 70 flights. The mission trajectory comprises waypoints defined to record autonomous test flights. This specific test case involves an obstacle avoidance scenario, employing Arducopter flying at an altitude of 80 meters. Its primary mission objective revolves around site surveillance while skilfully circumventing a towering obstacle, a building standing tall at a height of 110 meters. Our research endeavours comprised ten distinct locations, each carefully chosen to conduct the test flight experiments. At each location, a total of seven flights were executed, featuring six different sensors failure scenarios, coupled with one essential normal flight without any failure. This approach allowed us to explore and analyse the UAV's behaviour under various critical conditions.

### 3.2 Test Setup

**Fig. 5:**
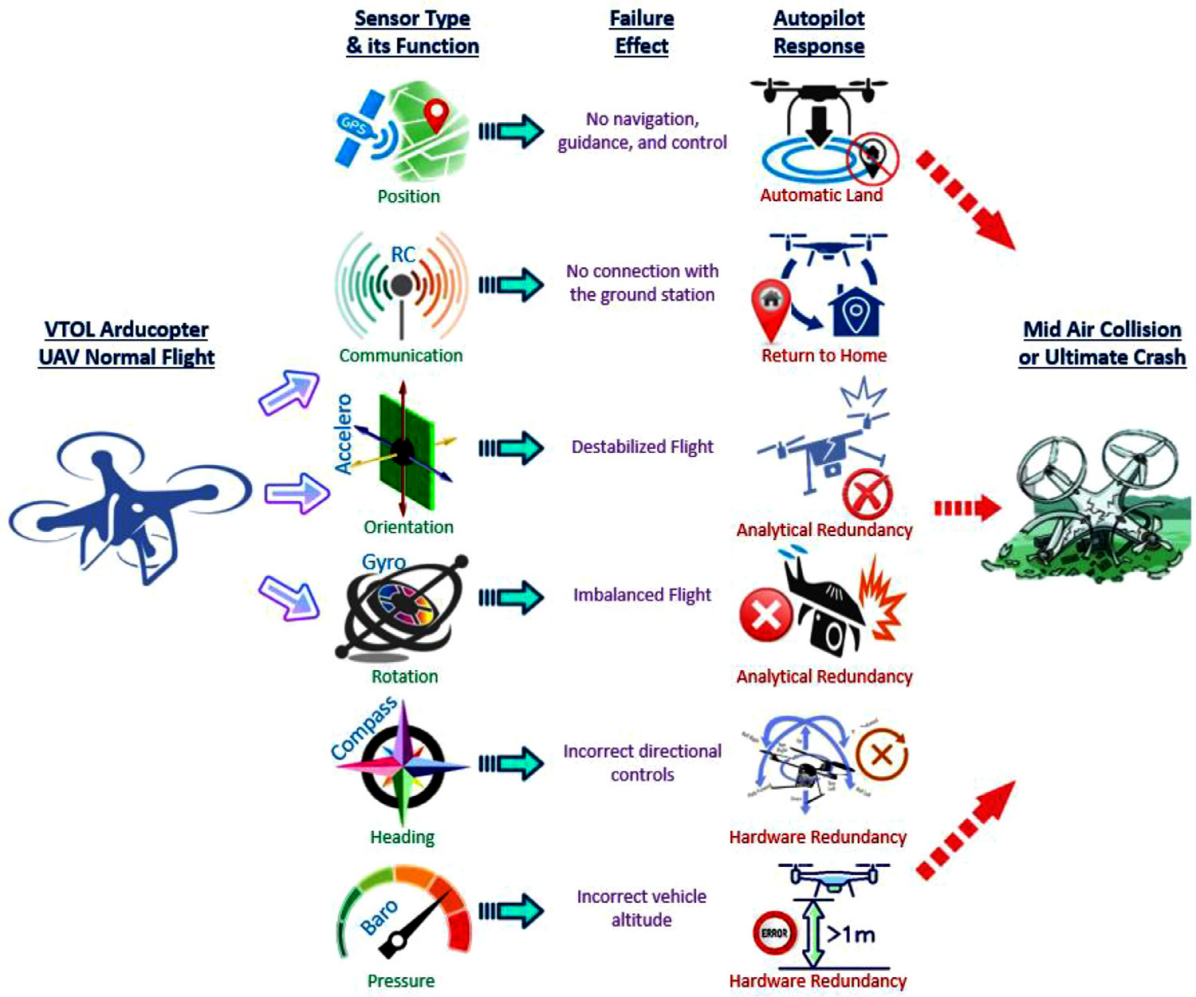
The unified protocol of test flights, showcasing normal flight initiation, selected sensors along with their respective functions, potential ramifications of sensor failures, the autopilot's responsive actions to address these failures, and the ensuing outcomes in the absence of successful mitigation strategies.

The execution of our test flights was conducted through a core functionality of Mission Planner, named *plan*. To create diverse missions for each of the ten test locations, we defined numerous waypoints, tailoring each mission's trajectory to its specific objectives. Subsequently, these missions were uploaded to the Arducopter firmware, ensuring precise execution during the flights. Each flight sequence commenced by setting the mode to guided, followed by the vehicle's arming, and issuance of the takeoff command, which elevated the UAV to the desired altitude. Transitioning the ArduPilot to auto mode, the UAV started the execution of the loaded mission, systematically traversing the predefined waypoints in a predetermined order. Each location's initial flight was set to complete the mission under normal conditions, without any failures, serving as baseline for comparison. Subsequent flights, however, were tactically interrupted by explicitly issuing sensor parameter failure commands through MAVProxy, utilizing MAVlink communication protocols. Consequently, the UAV was observed to navigate through the remaining waypoints, attempting to complete the mission despite the injected failures, meanwhile recording the pre-failure and post-failure flight data. To simulate real-world scenarios, the failure time was always randomized, adding the realism of the data generated during these test flights. This well-structured approach enabled us to present a unified protocol for UAV sensory data generation through injected failures in autonomous test flights. For a comprehensive overview of this unified protocol, refer to Fig. 5.

The analysis of failures was facilitated through the utilization of dataflash logs, recorded during the autonomous flight and stored in the onboard memory of the Arducopter autopilot controller. After each flight, these logs were downloaded for comprehensive examination. To replicate and evaluate the sensors' failure scenarios, we engineered SITL parameters. By configuring their parametric values, we could effectively simulate specific failure scenarios tailored to the requirements of each test case. For example, to assess the ArduPilot response when facing a GPS failure during the flight, we sent the command *param set SIM_GPS_DISABLE 1* via MAVLink from the GCS. As a result, the *Status* flag within the onboard log message *GPS* underwent a significant change, transitioning from 6 to 1. In response to this simulated GPS failure, our Arducopter automatically transitioned to a failsafe state, promptly activating the land mode, and executed a vertical landing at its current position on the ground. Similar sort of arrangements were made for the rest of the sensors' failure test scenarios.

### 3.3 Failure Types

We selected six representative sensors to demonstrate our failure test scenarios. Sensor types along with their intended purpose during the flight and failure effects are elaborated in Table 2.

**Table 2:** Sensors types, their function during the flight along with failure consequences.

No.	Sensor	Purpose	Failure Effect
1	GPS	To determine position of the UAV during flight.	No positioning information results no navigation, guidance, and control.
2	Remote Control	To send radio signals from ground control station to the UAV.	No connection link results no communication with the GCS.
3	Accelerometer	To measure orientation of the UAV for stabilization during the flight.	No linear acceleration information results flight destabilization.
4	Gyroscope	To measure the UAV's rate of rotation for balancing in the air.	No rotational acceleration information results flight imbalance.
5	Compass	To determine heading information for directional controls of the UAV.	No heading information results incorrect directional navigation.
6	Barometer	To measure atmospheric pressure for maintaining height of the UAV.	No altitude information results incorrect flight dynamics calculation.

## Limitations

### Simulation environment

The BASiC dataset is exclusively derived from simulations conducted in a SITL environment using the ArduPilot platform. As such, the dataset may not fully capture the complexities and variations present in real-world flight scenarios, potentially limiting its generalizability to actual operational conditions. Future iterations may feature a combination of simulated and actual flight scenarios, broadening the dataset's representativeness.

### Single platform dependency

The dataset relies on the ArduPilot platform only, which, while versatile, may limit the generalizability of findings to other autopilot systems like PX4 and openpilot. Future variations in firmware and control algorithms among different platforms may yield diverse responses to sensor failures.

### Individual Sensor Focus

In our current work, we intentionally focused on a detailed exploration of individual sensor failure scenarios. Future enhancements may address situations involving multiple simultaneous sensor failures, to highlight the challenges faced by UAVs in practical scenarios.

## Ethics Statement

The authors of this study support the ethical standards in line with the requirements for publication. It is pertinent to emphasize that the research conducted does not involve human subjects, animal experiments, or data collected from social media platforms. In adherence to the principles of transparency and scientific progress, the Biomisa Arducopter Sensory Critique (BASiC) dataset is now made publicly available for research and development purposes. To ensure proper acknowledgment, researchers utilizing this dataset are requested to adhere to the established citation rules.

## Credit Author Statement

The authors of this study have collaboratively and equally contributed to the generation of the Biomisa Arducopter Sensory Critique (BASiC) dataset. Their shared commitment to this endeavor underscores their mutual dedication to the research and development process. It is essential to highlight that there are no conflicts of interest between the authors, ensuring a harmonious and impartial approach in the creation of this dataset.

## Data Availability

Biomisa Arducopter Sensory Critique (BASiC) Dataset (Original data) (Zenodo) BASiC Dataset (Raw Data) (Original data) (Zenodo)
